# A combined imaging, deformation and registration methodology for predicting respirator fitting

**DOI:** 10.1371/journal.pone.0277570

**Published:** 2022-11-11

**Authors:** Silvia Caggiari, Bethany Keenan, Dan L. Bader, Mark N. Mavrogordato, Kathryn Rankin, Sam L. Evans, Peter R. Worsley

**Affiliations:** 1 Clinical Academic Facility, School of Health Sciences, University of Southampton, Southampton, United Kingdom; 2 School of Engineering, Cardiff University, Cardiff, United Kingdom; 3 μ-VIS X-ray Imaging Centre, Faculty of Engineering & Physical Sciences, University of Southampton, Southampton, United Kingdom; 4 Mechanical Engineering Department, Faculty of Engineering & Physical Sciences, University of Southampton, Southampton, United Kingdom; Universiti Sains Malaysia, MALAYSIA

## Abstract

N95/FFP3 respirators have been critical to protect healthcare workers and their patients from the transmission of COVID-19. However, these respirators are characterised by a limited range of size and geometry, which are often associated with fitting issues in particular sub-groups of gender and ethnicities. This study describes a novel methodology which combines magnetic resonance imaging (MRI) of a cohort of individuals (n = 8), with and without a respirator in-situ, and 3D registration algorithm which predicted the goodness of fit of the respirator. Sensitivity analysis was used to optimise a deformation value for the respirator-face interactions and corroborate with the soft tissue displacements estimated from the MRI images. An association between predicted respirator fitting and facial anthropometrics was then assessed for the cohort.

## Introduction

Since the outbreak of the COVID-19 pandemic, respirators of the N95/FFP3 type have been widely used to protect healthcare workers and patients from the transmission of the virus. Although their use minimises the risk of infection, clinical personnel have been required to wear respirators for prolonged periods, with frequent reports of fitting issues and discomfort being cited [1]. Indeed, as a consequence of the prolonged use of these respirators, adverse skin reactions including redness, indentation, and pressure ulcers (PUs) have been documented [2].

In order for these devices to provide efficient filtration while minimising air leakage, it is essential that FFP3 respirators fit tightly against the face, creating an airtight seal around the nose and mouth. Respirators are often selected following either a qualitative or quantitative fit testing procedure to identify the most appropriate device for the user [3]. However, current respirator designs rely on a panel of anthropometric features derived from a predominantly white male cohort (e.g., EN-149 standards), providing a limited range of size, geometries and materials. Accordingly, to compensate for a poor fit, respirators are typically over-tightened resulting in high non-uniform pressures [4], particularly at bony locations of the face e.g. nasal bridge and cheeks. In a recent study [5] the authors demonstrated an association between the interface pressure exerted on the nasal bridge and facial anthropometrics, with narrow nose shapes subjected to higher pressures. This highlights the importance of an effective fitting process prior to use, and the potential issues where face geometry do not conform with the design and material of the respirator.

To date, there is a paucity of technologies to support the fitting of medical devices, such as respirators. However, Computer Aided Design (CAD) image analysis and registration has been used for other devices such as prosthetic socket designs [6]. This has provided the means to optimise the conditions at the interface at the residual limb for load transfer and to protect vulnerable skin sites [7].

Adopting a modified algorithm [7], a number of distinct parameters indicative of the goodness of fit (GoF) for non-invasive ventilation (NIV) masks were previously identified, derived from surface scans of faces and the CAD geometry of a commercial mask device [8]. However, the authors restricted their analysis to Caucasian face shapes, using a rigid body registration. A similar approach has been used by Visscher, White [9] to evaluate the fit of NIV respiratory masks in a cohort of paediatric patients. The findings from both these studies were limited by omitting the simulation of deformation in both the respirator and face, with the rigid body assumptions restricting the known conformity when the device is in-situ. Indeed, most FFP3/N95 respirators have a highly flexible materials to adapt to different face geometries, where rigid body alignment would be inappropriate.

A recent study reported the development of a web-application for the prediction of respirator size and model for the individual user [10]. However, this was performed against the facial anthropometrics panel of the National Institute for Occupational safety and Health (NIOSH) ISO digital head forms (NIOSH). Each head form is symmetric and represents the facial size and shape distribution of a current US respirator user [11], which does not reflect the healthcare user population, with females showing the lowest prediction accuracy [12]. The prediction of fit was also not corroborated with fit test outcomes from standard test methods, which limits the translation of this approach. Recently, two studies have reported the development of intelligent workflows, involving 3D imaging and numerical simulations to validate custom-fit respirators [13, 14]. However, the fit of these devices was not validated *in vivo* and their performance against commercial respirators is yet to be assessed. In addition, the methodologies used are based upon semi-automated modelling with manual input to align the respirator geometry to the individual scan [13].

There is a critical need to develop intelligent platforms to support efficient respirator fitting of current commercial respirators and characterise the goodness of fit in an objective manner. This provides the motivation of the present study which aimed to develop an automatic methodology to determine the GoF of a single use commercial respirator, using a combination of 3D magnetic resonance imaging (MRI), deformation, and registration algorithms. It will incorporate a series of deformable respirator-face interactions and corroborate these interactions with the soft tissue displacement as quantified with the MRI technique.

## Materials and methods

The study was conducted in three distinct phases. The first phase was to capture the geometry of face shapes on a cohort of volunteers (n = 8) pre- and post-respirator application using MRI. In addition, micro-focus computed tomography (μCT) was used to image the internal geometry of a deformed respirator, segmented using the image processing software, ScanIP (Simpleware, Synopsys, USA). In the second phase, the MRI face data pre- and post-respirator application were used to estimate the soft tissue deformation. In parallel, a sensitivity analysis on a deformation factor for the respirator-face interactions predicted using a modified version of the python module Ampscan was conducted [7, 8]. In the final phase, the optimal respirator deformation was established, and the respirator predicted fitting was assessed against the participant’s facial anthropometrics. The study follows the guidelines outlined in the Declaration of Helsinki.

### Participants

Eight healthy participants (four males and four females) were recruited from the local community under institutional ethics (EC.21.01.12.6256A). They were aged between 22 and 36 years (mean = 31) with an average height and weight of 1.70 ± 0.09 m and 74.5 ± 15.9 kg, respectively. The corresponding BMIs ranged between 19.8 and 29.5 kg/m^2^. Six participants were White Caucasian and the remaining two were Asian of mixed ethnic background.

### Magnetic resonance imaging of tissue deformation during respirator application

A high-resolution 3D MRI sequence [15] was used to identify the volunteers’ anatomical face geometry in an unloaded (respirator not in situ) and loaded (respirator in situ) state. Each individual was scanned in supine using a 3 Tesla system (Siemens Magnetom Prisma) with a 64-channel head and neck coil. A T1-weighted (MPRAGE) sequence was used for all scans (0.7 mm iso, repetition time: 2100 ms, echo time 3.94 ms, flip angle 8°, acquisition time: 9 mins 30 secs). Ethical approval was granted by the Cardiff University School of Psychology Ethics Committee (EC.21.01.12.6356A). Written consent was obtained from each participant prior to testing. They then donned a commercial respirator (Easimask FSM18, UK), according to manufacture guidelines for application, and a facial scan was captured to characterise the loaded tissue state. The respirator has been reported as “MRI safe”, with no ferromagnetic components [16], and an MRI safety analysis was carried out prior the commencement of the study [15].

The facial scans for each participant were segmented and the surface geometry was converted to a binary.stl file. These were imported into Meshlab [17] where subjected to a cleansing process to eliminate unnecessary elements, and 90° rotation to the x-y plane was performed prior loading into the Ampscan module.

### Respirator geometry

A model of a single use FFP3 respirator, characterised by an un-valved semi-rigid shell, was purposely chosen as it represents a commonly procured model in the UK. Its internal geometry was captured following its application to a 3D printed head model corresponding to the small NIOSH head form [11]. Briefly, a phantom model corresponding to the small NIOSH head form was initially produced [15]. This contained an internal grid which would cause imaging artefacts and a limited contrast between the respirator geometry and the head material, limiting to capture the respirator seal geometry when in situ. The phantom head model was then μCT scanned at the μ-VIS X-ray Imaging Centre, with a custom 225/450 kVp Hutch scanner (Nikon Metrology, UK), and a surface mesh.stl file was generated. This was prepared using Ultimaker Cura 3.7 software for fused filament fabrication 3D printing in black polylactic acid (PLA), with 0.3 mm layer height, 0.4 mm line width, 0.8 mm wall thickness and no infill. The model was positioned with the coronal plane perpendicular to the print bed, and 3D printed using a 3D printer Delta WASP 2040 (WASP, Italy).

The respirator was then placed on the printed head model according to the manufacturer guidelines and subjected to a deformation against the rigid printed model (rigid state). The model with and without the respirator were μCT scanned. Subsequently, the reconstructed volume of the former was then segmented using ScanIP and the 3D mesh.stl files of the entire geometry and the internal surface of the respirator were exported.

### Ampscan module to predict respirator alignment and deformation

**Fig 1:**
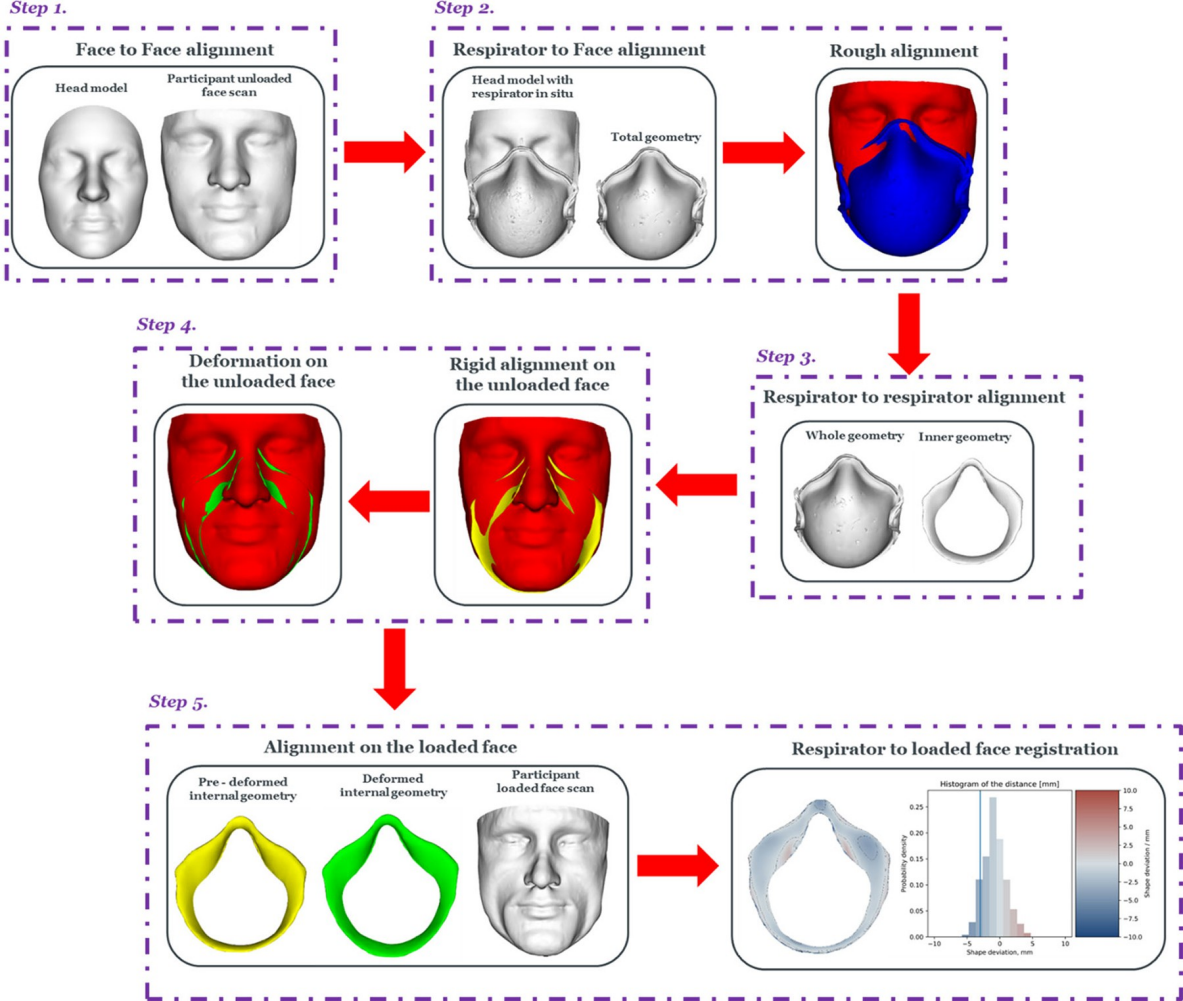
Modified Ampscan workflow. Schematic depicting the processes involved in predicting respirator alignment and deformation using Ampscan. This included a multi-step alignment and registration procedures.

A modified version of the python module Ampscan [7] was used to align, deform and register the internal geometry of the respirator onto the face geometry of each participant. This module was previously adapted to evaluate the goodness of fit of NIV [8], which was further modified to accommodate the process associated with the deformable respirator-skin interface. This consisted of a series of alignment and deformation processes as illustrated in Fig 1.

The sequence of alignment and registration steps are detailed below:

**Step 1 –Face to face alignment.** Orientation of participant’s facial scans in both unloaded and loaded state with respect to the target NIOSH head through an Iterative Closest Point (ICP) algorithm.

**Step 2 –Respirator to face alignment.** ICP algorithm was applied to achieve the orientation of the respirator’s whole geometry with respect to the target NIOSH head with respirator in situ. This created a rough alignment of the respirator onto the individual face.

**Step 3 –Internal respirator alignment.** Orientation of the respirator internal geometry was achieved by using the reference model from Step 2.

**Step 4 –Respirator- alignment and deformation to unloaded face.** A combination of rigid and non-rigid ICP algorithms [18] was applied to align and deform the internal respirator geometry to the unloaded face. A sensitivity analysis was conducted on a range of deformation values (δ values), where δ_1_ results in a highly conformed surface of the respirator with respect to the unloaded face whereas δ_8_ has a near rigid deformation.

**Step 5 –Respirator alignment and registration onto the loaded face.** The respirator internal geometry, in its rigid and deformed state from step 4, was then aligned and registered with respect to the loaded face geometry. A registration shape was generated depicting the distance between each respirator vertex and its nearest neighbour on the face, using a colour contour plot and a histogram representing the probability density of these distances (Fig 1).

### Quantification of soft tissue deformation from MRI scans

**Fig 2:**
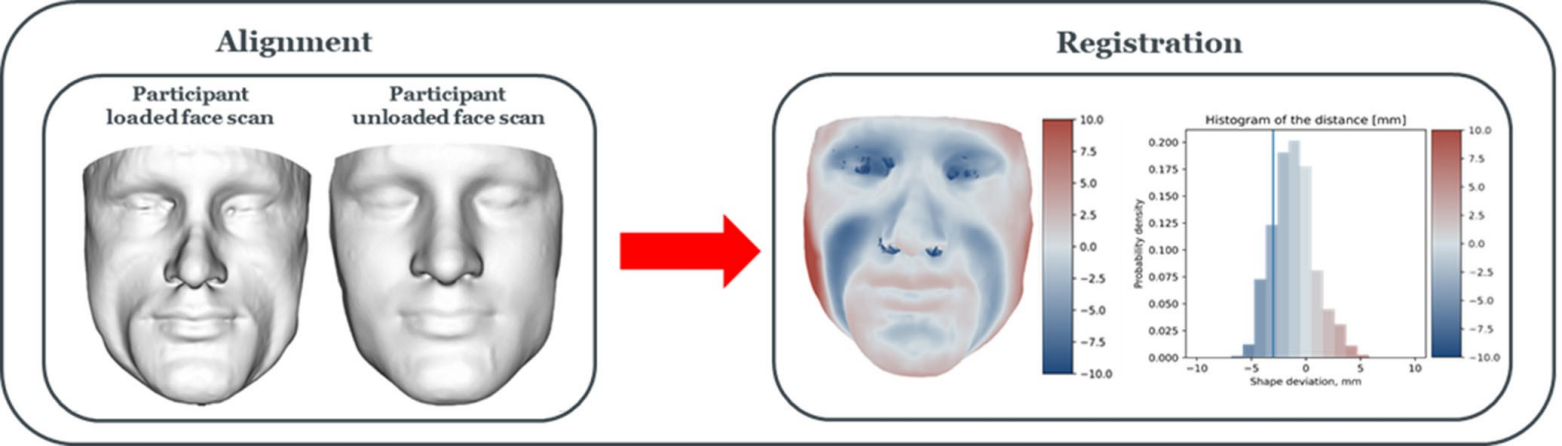
Loaded-to-unloaded face registration. Schematic depicting the alignment and registration processes involving facial scans in both loaded and unloaded states, to quantify the tissue deformation during respirator application. Distances between the vertices from each scan is represented as colour contour plot and histogram.

The face geometries estimated from the MRI scans during both unloaded and loaded test conditions were aligned and compared to quantify the tissue deformation during respirator application. A registration shape was then produced and the distance between the vertices from each scan was evaluated. This distance was depicted by a colour contour plot and represented as histogram of probability distribution (Fig 2). Displacement values exceeding -3mm and 0mm represent the threshold for skin indentation and gapping, respectively. For all individuals, the lower and upper 95^th^ percentile of the distribution were calculated, to represent the positive and negative soft tissue displacements that occurred following respirator application.

### Sensitivity analysis and corroboration of respirator deformation

The internal surface of the respirator was fitted to each volunteer’s face geometry using the fitting algorithm (Section 2.3) for all levels δ of deformation. The resulting frequency distributions of gapping and indentation were compared with those observed in the MRI sequences for each individual. Corroboration was performed through a series of steps designed to identify the respirator deformation (δ optimum) that conformed with the facial soft tissue displacement. This was achieved by evaluating the 95^th^ confidence interval (CI) of the respirator-face distance distribution for all δ values and the percentage area of the respirator which resulted in a face-to-face distance between -1 and 1 mm. δ optimum was considered to involve a combination of low CI value and high percentage conformity with the MRI deformation.

### Associations between GoF and anthropometric data

The respirator was then deformed with the optimum δ value on the unloaded facial scans. The resulting goodness of fit (GoF) parameters were used to evaluate associations with facial anthropometrics for all individuals. Three GoF parameters were then estimated which included the percentage of respirator surface which gaps from the skin (>0mm), the area which substantially indents the skin (<-3mm) and the corresponding remaining area which provides an adequate seal (range -3 to 0mm) [8]. In the latter case, the face comes into contact with the respirator, with a displacement that can be accommodated by the soft tissues, ensuring a safe fitting. From each unloaded facial scan, five facial anthropometrics were estimated, namely the lower 1/3 facial height, alar and bio-ocular width, and the dorsal nasal length among those more frequently used in other studies [19]. For each anthropometric, point to point distances were manually selected by the same investigator (SC), using corresponding landmarks on the surface scans. These were used to evaluate associations with predicted GoF values from optimised respirator fitting. Facial anthropometric data were examined for normality using Shapiro–Wilk tests, and parametric descriptors were found to be appropriate. Subsequently, Pearson correlation was used to evaluate associations, with the statistical significance level set at 5% level (p ≤ 0.05).

## Results

### Soft tissue deformation from MRI scans

**Fig 3:**
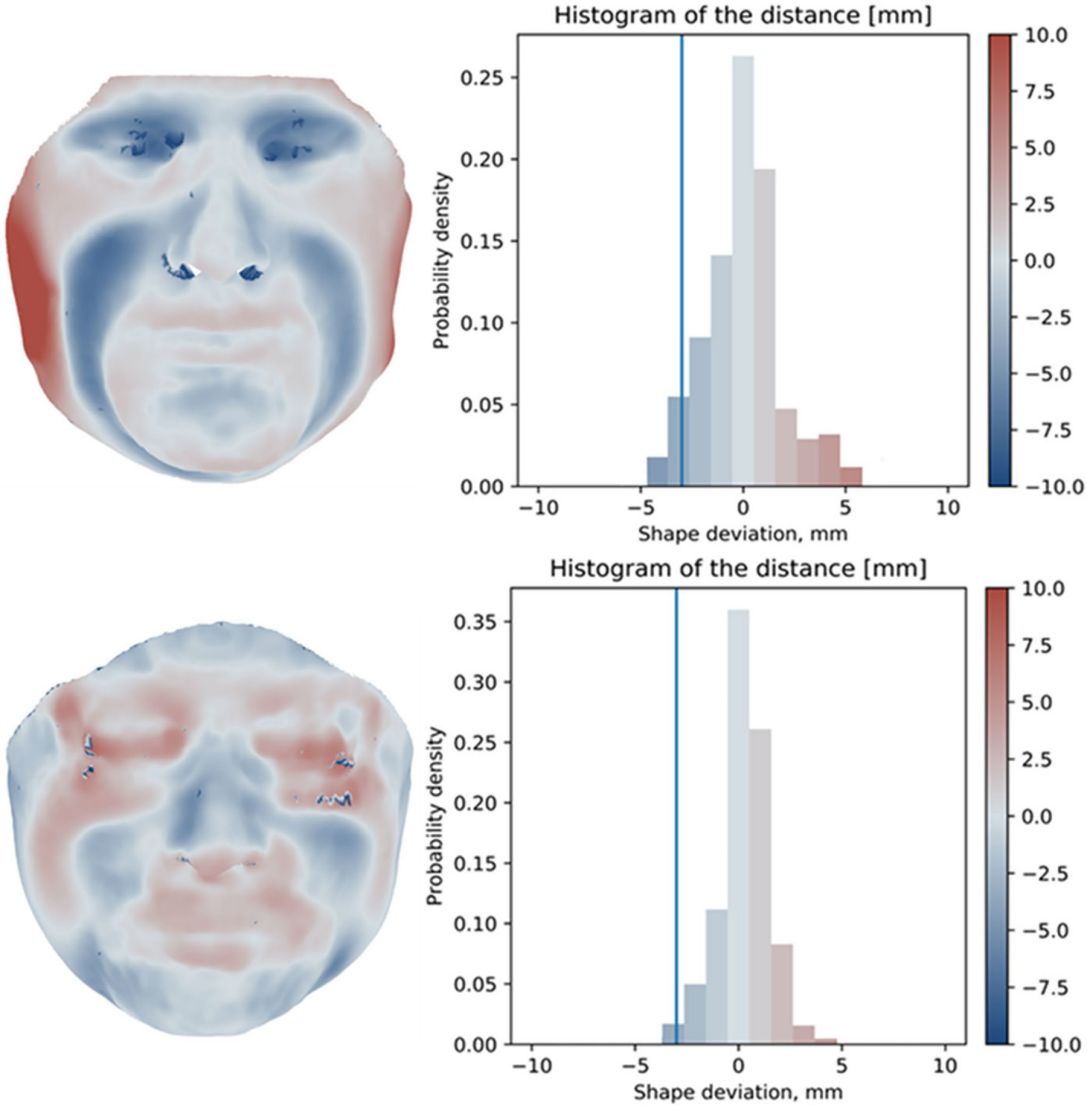
Characterisation of soft tissue deformation. Loaded to unloaded face registration and distribution of distances for two participants.

An exemplar of loaded face to unloaded face registration and the distribution of distances are presented in Fig 3, for two individuals. The extremes of the histogram represent the 95^th^ percentile values, which indicate the positive and negative displacements of soft tissues that occurred following respirator application. It is possible to observe that participant #1 (top) showed a displacement of approximately ±5mm, indicating a high degree of indentation of the respirator and a resulting positive soft tissue displacement. By contrast, participant #2 (bottom) showed a CI ranging from -3.9 mm to 4.4mm.

Findings across the cohort revealed that the negative soft tissue displacement ranged between -3.2 mm to -6.2 mm (mean = -4.59 ± 0.93 mm), indicating a high degree of indentation of the respirator onto the face of the individuals. By contrast, a positive displacement ranged between 3.1 mm and 6 mm (mean = 4.39 ± 1.01 mm), which occurred in the majority of the individuals on the lateral side of the face. Close examination of the individual data reveals that those individuals with the highest negative displacement i.e., ID#3 and #8, also demonstrated the highest positive displacement.

### Sensitivity analysis to determine optimum respirator deformation

**Fig 4:**
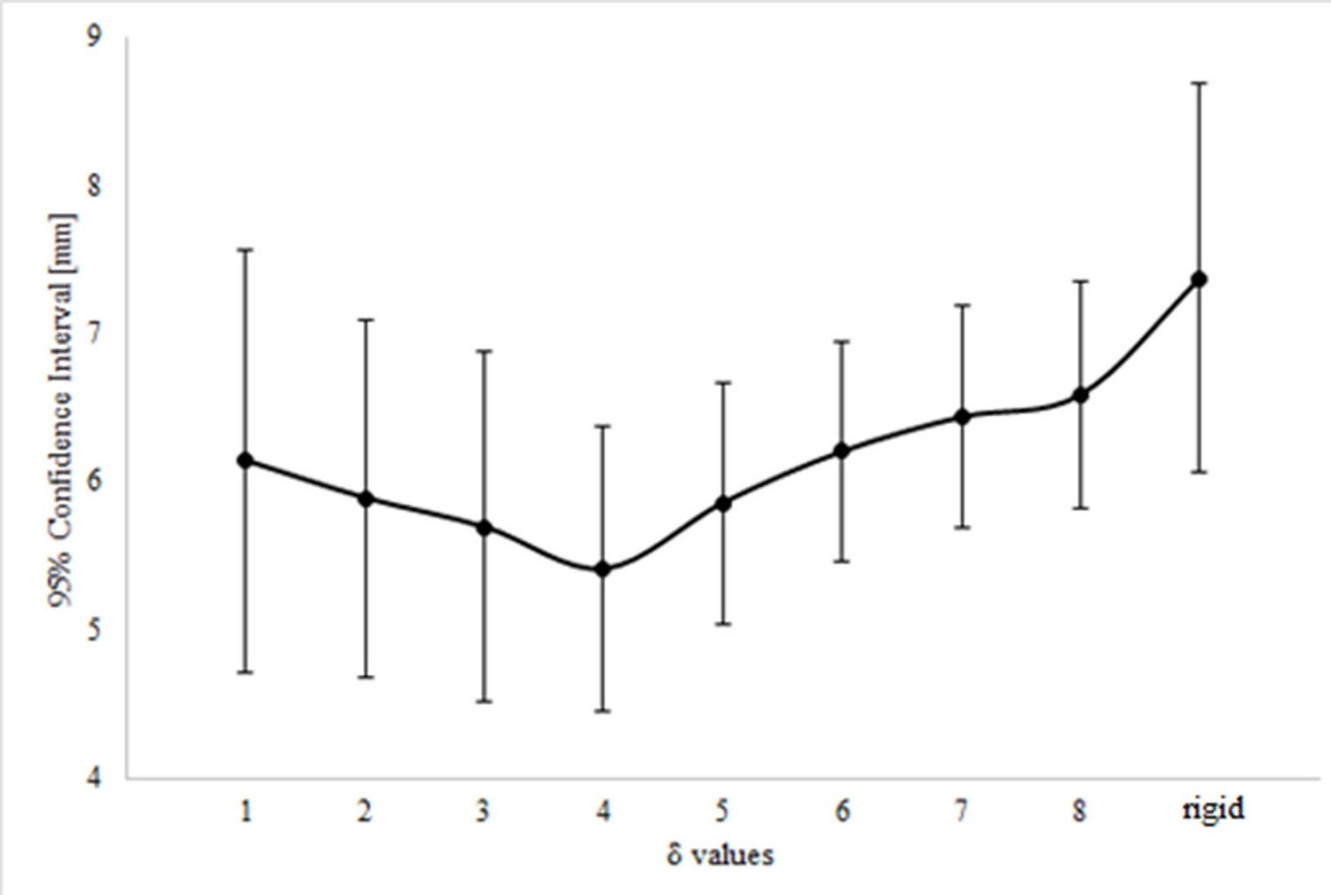
Sensitivity analysis on respirator deformation levels. 95% CI of the distance distributions resulted from the respirator-loaded face registration, with the respirator deformed with different levels δ of deformation.

The sensitivity analysis revealed a high degree of inter-subject variability, depicted by the large standard deviation for the 95% CI, for each of the δ deformation values (Fig 4). This was particular evident at the extreme deformations i.e., δ_1_ and δ_8_. The lowest variability was generally associated with the δ_4_ deformation (CI mean value = 4.9 mm, range 3.8–6.5 mm). By contrast, the registration of the respirator in its rigid state had a CI value of 6.9 mm (range 5.7–9.7 mm).

Results on the percentage area of the respirator which resulted in a high degree of conformability for each of the loaded participant face shapes (± 1 mm) are summarised in Table 1. It was clear that for most of the participants (7/8) the respirator in its rigid state resulted in the lowest conforming percentage area. The higher percentage area values were associated with δ_2–4_ values (bold), although the degree of optimum conformability varied between subjects (53% and 78%).

**Table 1:** Percentage respirator area resulting in a high degree of conformability to loaded face shapes, for all deformation values.

*Deformation values*	*ID #1*	*ID #2*	*ID #3*	*ID #4*	*ID #5*	*ID #6*	*ID #7*	*ID #8*
*δ*_*1*_	64.5	68.2	52.2	56.4	54.9	50.5	65.4	54.8
*δ*_*2*_	**66.5**	70.5	52.7	58.2	55.0	51.4	70.7	57.2
*δ*_*3*_	65.7	**73.8**	**53.3**	62.1	**56.2**	**53.1**	**72.5**	62.2
*δ*_*4*_	61.7	68.1	47.9	**67.6**	55.5	49.6	56.3	**73.3**
*δ*_*5*_	53.9	53.7	46.4	63.4	53.7	42.9	39.2	71.8
*δ*_*6*_	48.1	46.5	45.7	56.8	53.2	39.9	32.8	67.1
*δ*_*7*_	45.1	43.9	46.0	54.1	52.7	38.1	30.7	63.6
*δ*_*8*_	43.1	41.9	45.8	52.6	52.3	37.0	29.9	62.3
*rigid*	30.5	36.3	44.7	45.8	51.5	35.8	20.8	55.3

Percentage respirator area which resulted in a distance from the loaded face between -1 and 1 mm, for the deformations δ_1–8_. The highest percentage values for each individual are in bold.

### Association between respirator fitting and anthropometric measures

**Fig 5:**
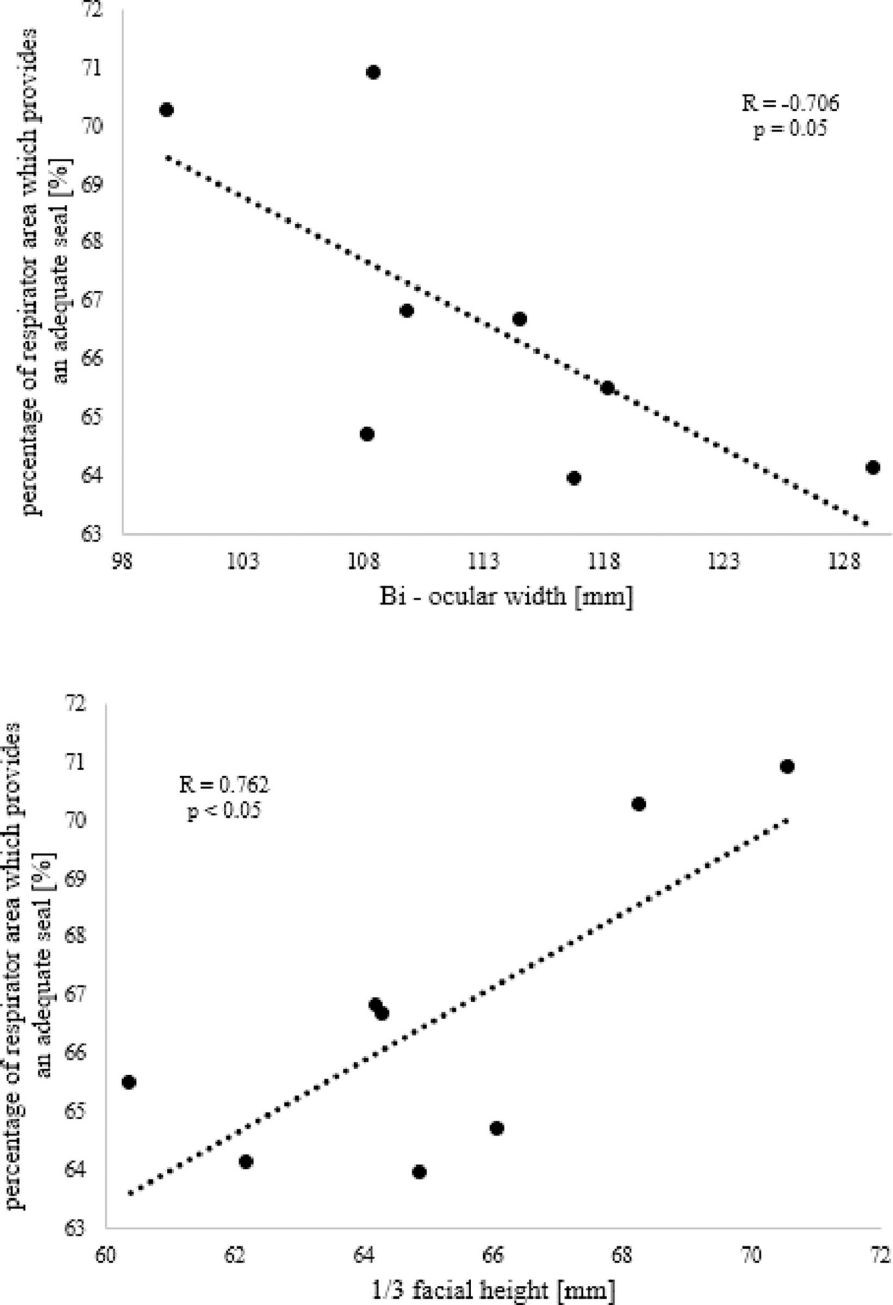
Effect of facial anthropometrics on respirator area providing an adequate seal. Relationship between the percentage of respirator area which provides an adequate seal [%] and bi-ocular width (top) and 1/3 facial height (bottom).

A subject-specific *δ optimum* value (derived from Table 1) was used to deform the respirator onto the unloaded face and examine the association between conformability and the individual facial anthropometric features. Results revealed that the percentage of respirator area which provides an adequate seal ranged between 63% and 71%. When these values were compared against the facial anthropometrics, a statistically significant association (p<0.05) was found against the bio-ocular width and the 1/3 facial height of the participants, as presented in Fig 5.

## Discussion

The present study describes a novel methodology which combines 3D MRI imaging, deformation, and registration algorithms to characterise deformable respirator-face interactions of a single use commercial respirator of FFP3 type (Fig 1). μCT was used to capture the geometry of the respirator, which allowed to have high resolution 3D image. The algorithm was optimised and corroborated against MRI data of eight volunteers who were scanned with and without the respirator in-situ (Figs 2 and 4). To the best of the authors knowledge, no previous studies have investigated and characterised the respirator deformation to support an intelligent and efficient fitting process. Findings revealed a subject-specific *δ optimum*, which by implication highlight the diversity in the respirator fit against the individuals face. Goodness of fit parameters derived from this optimum deformation then revealed significant associations with facial anthropometrics, which require further investigation.

MRI analysis revealed a high degree of skin and soft tissue deformation with the respirator in-situ (Fig 3), exceeding in all the cases 3mm from baseline unloaded images. This has previously been used as a threshold for skin indentation whereby tissue could be put at risk of damage [8]. This is based on the established sigmoid curve of soft tissue deformation and exposure time implicated in pressure ulcer development [20]. The deformation estimated in the MRI data can be associated with the morphology of the skin and soft tissues of the face. Shah and colleagues [21] used a mechanical indenter device on specific facial landmarks at threshold levels of both discomfort and pain, on a cohort of healthy volunteers. The authors reported that the cheeks were subjected to the highest deformation at both thresholds, as opposed to the nasal bridge which was subjected to the lowest deformation [21]. Indeed, it is acknowledged that this latter location has minimal soft tissue coverage where skin is overlying rigid bone and cartilage sub-dermal structures. Accordingly, it represents a site of limited tolerance to mechanical loads, commonly subjected to respirators-related facial injuries [22]. The facial colour contour plot showed darker tones of blue approximately at the cheeks’ location, indicating a higher degree of respirator indentation, as opposed to the nasal bridge which was characterised by lighter tones (Fig 3).

Several studies have identified facial dimensions as predictors of fitting outcomes [23–26]. In particular, a few studies [23, 27] reported facial height, alar width and dorsal nasal length to correlate to fit factors with these measures statistically significant different in individuals who passed and failed the fit test [27]. However, there is a lack of reports which have correlated facial measurements with objective GoF parameters.

The present study investigated the association between the percentage of respirator area which provided an adequate seal and a range of facial measurements. Results revealed a statistically significant correlation (p ≤ 0.05) with bio-ocular width and 1/3 facial height (Fig 5), with the higher percentage of adequate seal associated with narrower and longer faces. By contrast, no statistically significant association was observed when respirator’s adequate seal was compared against, alar width, and dorsal nasal length. Choice of 1/3 facial height is justified by the fact that 3D facial images of the participants did not include the total facial height (Fig 1).

A recent study [13] used the maximum distance measured at respirator-face interface as objective indicator of GoF of a custom-fit device. Results reported a mean value of 2.03 mm (±0.75) across the cohort of participants (n = 205), with females and individuals from ethnic minority groups showing the lower distance. Although our findings revealed a similar mean distance of 2.86 mm (±0.75), the limited number of volunteers in the present study limits comparisons between gender and ethnicity.

In a recent study the authors also demonstrated an association between the interface pressure exerted on the nasal bridge and the alar width, with narrow noses shape subjected to higher pressures [5]. This highlights the importance of integrating respirator-face biomechanical interactions, intelligent algorithm for fitting prediction, and anthropometric measures to support efficient respirator fitting and characterise the GoF to individual face shapes in an objective manner.

Intelligent algorithms are increasingly being developed to characterise the interactions at the face-respirator interface and customise the design of these devices [13, 14, 28, 29]. Recent studies [13, 14] reported the development of intelligent platforms to generate a custom-fit respirator, across sub-categories of individuals demographics [13]. However, Degueldre, Borduas [14] based the prediction of fit on the evaluation of the contact pressures, and used costly computational numerical simulations, e.g., Final Element modelling. By contrast, Li, Tan [13] reported the development of a semi-automatic process where 3D facial scans were combined with a CAD modelling to evaluate the fit of a custom-based respirator. This implied a manual alignment of the respirator geometry onto the face. In contrast to these studies, the present analysis has included an optimisation and corroboration analysis to known tissue deformations estimated using MRI. This critical step provides additional confidence in the predicted fitting and corresponding GoF values produced by the algorithm.

Despite optimisation of the fitting algorithm, the analysis still revealed a high degree of inter-subject variability in fitting outcomes and a high probability of gapping and indentation known to cause infection risk, and skin damage, respectively. Thus, collaboration with manufacturers is needed to identify new designs of respirators and create standards which accommodate face shapes of different genders and ethnicities. There is the need to support standard fitting methods with intelligent algorithms and characterise the GoF in an objective manner to provide individuals with safe and effective respiratory protective equipment.

## Limitations

The present study is clearly limited by its analysis of only one type of FFP3 respirator, which does not necessarily represent all respirator designs. The non-rigid ICP algorithm applied to deform the internal respirator geometry was limited in its ability to discriminate between different sections of the respirator which might have been subjected to different degree of deformation. Indeed, for each of the deformation values, all the respirator vertices were deformed with the same *δ*. Nonetheless, the *δ* values were conveniently chosen to represent different degree of respirator conformability with respect to the individuals face. A further limitation was the use of a relatively small cohort of healthy volunteers (n = 8) recruited from the UK, who were predominantly Caucasian and clearly do not represent the ethnic diversity reflective of the healthcare worker population. Indeed, further research is needed to investigate the fit of a wider range of respirator models on a larger and more diverse cohort [30]. In addition, individuals were scanned supine, a position which does not reflect postures adopted by healthcare workers during a working shift, but which does allow the deformation of the soft tissues to be captured.

## Conclusion

This study used a combination of 3D imaging, deformation, and alignment algorithms to characterise the fit of a single use commercial respirator. The respirator deformation was estimated with respect to soft tissues displacements from a ‘loaded to unloaded face’ registration, using 3D MRI scans. The results revealed a high degree of indentation of the respirator on the facial soft tissues. They also showed that specific facial features e.g., bio-ocular width and 1/3 facial height, were related to goodness of fit parameters. The development of an intelligent fitting software could be used to support respirator selection and identify new design features which can accommodate a diverse range of face geometries.

## References

[1] TUC. Personal protective equipment and women. 2017.

[2] Abiakam N, Worsley P, Jayabal H, Mitchell K, Jones M, Fletcher J, et al. Personal protective equipment related skin reactions in healthcare professionals during COVID-19. Int Wound J. 2021;18(3):312–22. doi: 10.1111/iwj.13534 33507634 PMC8013193

[3] Rollings L. FFP3 respirator face fit testing—what is it all about? British Dental Journal. 2020;229(2):112–4. doi: 10.1038/s41415-020-1850-x 32710057 PMC7380143

[4] Lei Z, Yang J, Zhuang Z. Headform and N95 Filtering Facepiece Respirator Interaction: Contact Pressure Simulation and Validation. Journal of Occupational and Environmental Hygiene. 2012;9(1):46–58. doi: 10.1080/15459624.2011.635130 22168255

[5] Caggiari S, Bader D, Foxell F, Pipe N, Couch S, Turner A, et al. Biomechanical and Physiological Evaluation of Respiratory Protective Equipment Application. Med Devices (Auckl). 2022;15:241–52. doi: 10.2147/MDER.S370142 35928220 PMC9343257

[6] Dickinson AS, Steer JW, Woods CJ, Worsley PR. Registering methodology for imaging and analysis of residual-limb shape after transtibial amputation. J Rehabil Res Dev. 2016;53(2):207–18. doi: 10.1682/JRRD.2014.10.0272 27148905

[7] Steer JW, Stocks O, Parsons J, Worsley PR, Dickinson AS. ampscan: A lightweight Python package for shape analysis of prosthetics and orthotics. J Open Source Softw. 2020;5:2060. 10.21105/joss.02060.

[8] Verberne JWR, Worsley PR, Bader DL. A 3D registration methodology to evaluate the goodness of fit at the individual-respiratory mask interface. Comput Methods Biomech Biomed Engin. 2020:1–12. doi: 10.1080/10255842.2020.1849156 33241703

[9] Visscher M, White C, Jones J, Cahill T, Jones D, Pan B. Face Masks for Noninvasive Ventilation: Fit, Excess Skin Hydration, and Pressure Ulcers. Respiratory care. 2015;60. 10.4187/respcare.04036. 26420902

[10] Adlouni Ma, Choksi D, D’Souza B, Richards ZI, Sims IV RK, editors. Image-Based Web Application for Respirator Sizing: Contactless Mask-Fitting During a Pandemic. 2022 Design of Medical Devices Conference; 2022.

[11] Zhuang Z, Bradtmiller B. Head-and-Face Anthropometric Survey of U.S. Respirator Users. Journal of Occupational and Environmental Hygiene. 2005;2(11):567–76. doi: 10.1080/15459620500324727 16223715

[12] Zhuang Z, Landsittel D, Benson S, Roberge R, Shaffer R. Facial anthropometric differences among gender, ethnicity, and age groups. Ann Occup Hyg. 2010;54(4):391–402. doi: 10.1093/annhyg/meq007 20219836

[13] Li S, Tan Y, Willis S, Bahshwan M, Folkes J, Kalossaka L, et al. Toward Mass Customization Through Additive Manufacturing: An Automated Design Pipeline for Respiratory Protective Equipment Validated Against 205 Faces. International journal of bioprinting. 2021;7(4):417. doi: 10.18063/ijb.v7i4.417 34805596 PMC8600309

[14] Degueldre L, Borduas J, Dion F, Laurin P, Castonguay A, Viens S-P, et al. Improving the Fit of Respiratory Face Mask through 3D Scanning, Finite Elements Analysis and Additive Manufacturing. Proc of 3DBODYTECH 2020 - 11th Int Conf and Exh on 3D Body Scanning and Processing Technologies, Online/Virtual. 2020;#33.

[15] Keenan BE, Lacan F, Cooper A, Evans SL, Evans J. MRI safety, imaging artefacts, and grid distortion evaluated for FFP3 respiratory masks worn throughout the COVID-19 pandemic. Clinical Radiology. 2022;77(8):e660–e6. doi: 10.1016/j.crad.2022.05.001 35654622 PMC9108088

[16] Information on MRI safety of FFP3 masks. University Hospitals of Birmingham NHS Foundation Trust (2020). https://COVID-19.sor.org/getattachment/Diagnostic-Radiography-FAQs/MRII/FFP3-Masks-MRI-safety-info-v3-1-24-Mar-20.pdf?lang=en-GB.

[17] Cignoni P, Callieri M, Corsini M, Dellepiane M, Ganovelli F, Ranzuglia G. MeshLab: an Open-Source Mesh Processing Tool 2008. 129–36 p.

[18] Amberg B, Romdhani S, Vetter T, editors. Optimal Step Nonrigid ICP Algorithms for Surface Registration. 2007 IEEE Conference on Computer Vision and Pattern Recognition; 2007 17–22 June 2007.

[19] Chopra J, Abiakam N, Kim H, Metcalf C, Worsley P, Cheong Y. The influence of gender and ethnicity on facemasks and respiratory protective equipment fit: a systematic review and meta-analysis. BMJ Glob Health. 2021;6(11). doi: 10.1136/bmjgh-2021-005537 34764145 PMC8587533

[20] Gefen A. Reswick and Rogers pressure-time curve for pressure ulcer risk. Part 1. Nursing Standard. 2009;23(45):64–8. doi: 10.7748/ns2009.07.23.45.64.c7115 19678520

[21] Shah P, Luximon Y, Luximon A. Measurement of soft tissue deformation at discomfort and pain threshold in different regions of the head. Ergonomics. 2022:1–16. doi: 10.1080/00140139.2022.2028016 35007469

[22] Worsley PR, Prudden G, Gower G, Bader DL. Investigating the effects of strap tension during non-invasive ventilation mask application: a combined biomechanical and biomarker approach. Med Devices (Auckl). 2016;9:409–17. doi: 10.2147/MDER.S121712 27942235 PMC5136364

[23] Manganyi J, Wilson KS, Rees D. Quantitative Respirator Fit, Face Sizes, and Determinants of Fit in South African Diagnostic Laboratory Respirator Users. Ann Work Expo Health. 2017;61(9):1154–62. doi: 10.1093/annweh/wxx077 29136414

[24] Han D-H, Choi K-L. Facial Dimensions and Predictors of Fit for Half-Mask Respirators in Koreans. AIHA Journal. 2003;64(6):815–22. doi: 10.1202/501.1 14674801

[25] Zhuang Z, Coffey CC, Ann RB. The effect of subject characteristics and respirator features on respirator fit. J Occup Environ Hyg. 2005;2(12):641–9. doi: 10.1080/15459620500391668 16298949

[26] Milosevic M, Kishore Biswas R, Innes L, Ng M, Mehmet Darendeliler A, Wong A, et al. P2/N95 filtering facepiece respirators: Results of a large-scale quantitative mask fit testing program in Australian health care workers. American Journal of Infection Control. 2022;50(5):509–15. doi: 10.1016/j.ajic.2021.12.016 34971710 PMC8767955

[27] Zhang X, Jia N, Wang Z. The relationship between the filtering facepiece respirator fit and the facial anthropometric dimensions among Chinese people. Ind Health. 2020;58(4):318–24. doi: 10.2486/indhealth.2019-0158 31787708 PMC7417508

[28] Dai J, Yang J, Zhuang Z. Sensitivity analysis of important parameters affecting contact pressure between a respirator and a headform. International Journal of Industrial Ergonomics. 2011;41(3):268–79. 10.1016/j.ergon.2011.01.007.

[29] Lei Z, Yang J, Zhuang Z. A novel algorithm for determining contact area between a respirator and a headform. J Occup Environ Hyg. 2014;11(4):227–37. doi: 10.1080/15459624.2013.858818 24579752 PMC4747037

[30] Solano T, Mittal R, Shoele K. One size fits all?: A simulation framework for face-mask fit on population-based faces. PLOS ONE. 2021;16(6):e0252143. doi: 10.1371/journal.pone.0252143 34133436 PMC8208573

